# A Mechanical Approach for Comparing Jaws in Fishes

**DOI:** 10.3390/biomimetics9040239

**Published:** 2024-04-16

**Authors:** Federica Trotta, Roberto Sandulli, Simone Cinquemani

**Affiliations:** 1 Mechanical Engineering Department, Politecnico di Milano, Via Giuseppe La Masa, 1, 20156 Milan, Italy; federica.trotta@polimi.it; 2 Department of Science and Technology (DiST), Consorzio Nazionale Interuniversitario per le Scienze del Mare (CoNISMa), Parthenope University of Naples, Centro Direzionale—Isola C4, 80143 Naples, Italy; roberto.sandulli@uniparthenope.it

**Keywords:** bio-inspired, four-bar mechanisms, linkage mechanism, robotics

## Abstract

This paper aims to propose an quantitative engineering approach to study and compare the jaw mechanisms of different marine species, considering essential mechanical parameters generally used to evaluate the performance of industrial linkage mechanisms. By leveraging these parameters, the paper demonstrates how the species’ characteristics and behaviors align with the findings of biologists, enabling a meaningful comparison that was not previously possible. Seven fish species from various families are chosen to maintain a generic approach.

## 1. Introduction

In recent years, the adoption of bio-inspired solutions in robotics has seen a significant increase. Engineers draw innovative ideas from observing animal behaviors, as they often encounter similar mechanical challenges [1]. Natural selection has driven animals and plants to adapt their bodies and behaviors for self-preservation, seeking the most optimal solutions. In the realm of bio-inspired engineering, analyzing biological solutions becomes a crucial step in developing new designs [2]. Examples include drawing inspiration from chameleons’ tongues [3,4,5], octopus arms [6,7,8], or insect mechanics [9,10]. Biologists prefer qualitative approaches with anatomical analyses and geographical distribution, focusing on the species’ behaviors such as diets, habitats, and more. On the other hand, engineers model living beings as mechanical systems, considering muscles as actuators, bones, and articulations as linkage mechanisms, etc. [11]. The divergence in methods and tools is evident, underscoring the necessity to adopt a quantitative mechanical approach that aligns specifically with their interests.

One fascinating area of study is focused on fishes. Researchers have analyzed and modeled the fins to investigate the high efficiency of motion of aquatic animals, enhancing the swimming capabilities of aquatic robots [12,13,14,15,16]. Additionally, an interesting field for innovative grippers is taking inspiration from some fishes possessing unique jaw mechanisms, such as the Moray eel’s double jaw design [17], the upper jaw protrusion of *Trachipterus altivelis* [18], or the mouth opening phase of *Clarias gariepinus* [19]. These mechanisms frequently vary between different families and within species belonging to the same family. Consequently, when examining large animal families, it becomes challenging to make meaningful comparisons that lead to identifying the optimal solution for a specific problem through biological analyses. The significant anatomical and functional differences among species make direct comparisons difficult.

This paper aims to propose an quantitative engineering approach to study and compare the jaw mechanisms of marine species, considering essential mechanical parameters like the mouth’s opening and closing geometrical speeds. By leveraging these parameters, the paper demonstrates how the species’ characteristics and behaviors align with the findings of biologists, enabling a meaningful comparison that was not previously possible, which is more comprehensible for engineers. Seven fish species from various families are chosen to maintain a generic approach.

The jaws are represented as linkage mechanisms, and to facilitate comprehension, the structures are transformed from spatial to planar models while ensuring accuracy [11]. This simplification effectively captures the jaw motions performed by each species. Additionally, the crucial links and constraints are emphasized to present the mechanisms as clearly as possible. By analyzing these structures, whose complexity varies depending on the species, some interesting parameters useful for the investigation are derived.

The results obtained from the kinetostatic investigation of the linkage mechanisms are extrapolated, and comparisons between species are derived for each mechanical parameter. Furthermore, a comparison is made using measured velocities of each species from the existing literature. This analysis not only presents the impact of the structure itself but also highlights differences arising from anatomy, particularly concerning the length of the muscles.

These results have been compared to the biological notions studied beforehand in order to confirm how the mechanical analysis aligns with the biological one. This ensures that no information is lost; instead, it is presented from an alternative viewpoint. This proves the validity of the proposed engineering approach, demonstrating its effectiveness in understanding and evaluating the jaw mechanisms.

## 2. Methods

A quantitative analysis of the behavior of fish jaws is the starting point for setting up a design of manipulators that are inspired by these biological systems. This approach not only enables comparative analyses, but also allows for a quantification of the performance of the different biological solutions. Specifically, the methodology presented in this paper derives from the meeting of biomechanical techniques established within the field, with those of functional analysis adopted in the world of automatic machines.

### 2.1. Mechanical Approach

From a biomechanical perspective, living beings can be represented as mechanical systems comprising rigid bodies interconnected through ideal joints and driven by external or internal forces, primarily exerted by muscles or resulting from interactions with the external environment.

To analyze the biomechanics of different species’ jaws, their skeletal structures have been simplified as linkage mechanisms [20]. In this context, rigid components of the jaw, such as bones, are treated as unyielding links capable of movement and rotation but not deformation. It is important to note that the bone’s shape is not significant from a kinetostatic perspective. Therefore, even intricate shapes are approximated using rigid straight beams. Similarly, the joints connecting the bones are represented as hinges. Muscles are considered as ideal actuators; when activated, they contract, causing movement throughout the kinematic chain. This modeling approach accurately represents the biomechanics of skeletal structures [11]. Additionally, all the jaw mechanisms of the analyzed fish can be depicted as 2D structures, implying that all links move within the same plane. This advantage simplifies computations and analyses. This approach is implemented since it is adequate for the purpose of the study presented here; however, in reality, the 3D motion is rather complex and the parts are not ideal. These aspects have to be taken into account if the goal is to reach a full biological understanding of the species.

Drawing on the mechanical analogy between biological structures and corresponding linkage structures, each mechanism has an input actuation that initiates movement in the first link (known as the driver). In this case, the input actuation corresponds to muscle contraction. The motion is then transformed and transmitted to the final link (called follower), typically representing the jaw. It is essential to recognize that different species employ distinct muscles for the opening and closing phases of jaw movement. As a result, these two phases must be analyzed individually since the mechanism’s topology changes when the input force/displacement is applied to different links.

To comprehensively assess the conduct of diverse species, we can overlay a biomechanical approach onto the functional analysis of articulated systems, drawing inspiration from practices employed in automatic machines. Presented below are several parameters that will facilitate the description and quantification of the behavior exhibited by various marine species.

**Figure 1:**
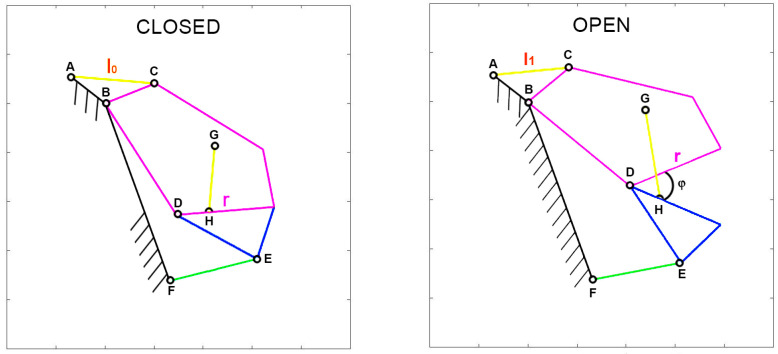
The upper jaw link is pointed out as *r*, the muscle length at rest is *l*_0_ and the contracted one is *l*_1_, *φ* is the jaw gape angle.

Given that various parameters are associated with the input actuation, special attention should be given to defining muscle contraction accurately. To facilitate comparisons between species with diverse shapes and sizes, muscle contraction is normalized with respect to a specific length. Two normalization methods are of particular interest. The first, known as “normalized contraction” (*ν*), considers the shortening of the muscle normalized over the maximum length of the upper jaw in the respective species. This normalization choice arises from the need to compare different organisms that vary significantly in size. By using this normalization, it becomes possible to analyze how the gape angle changes in response to length variations of the input muscle, independent of the species’ dimensions. Consequently, normalized contraction is computed as the difference between the length of the muscle at rest (*l*_0_) and the length of the contracted muscle (*l*_1_) over the length of the upper jaw (*r*). Refer to Figure 1 for illustrations of these characteristic lengths.

(1) $$v=\frac{l_{0}-l_{1}}{r}$$

Another valuable normalization involves expressing the length reduction of the muscle ($l_{0}-l_{1}$) as a percentage of its initial length at rest ($l_{0}$). This is essentially a “percentual contraction” (*ρ*) of the muscle.

(2) $$\rho =\frac{l_{0}-l_{1}}{l_{0}}\cdot 100$$

To elucidate the mechanism’s ability to transfer motion from its input (muscle contraction) to its output (jaw motion), several kinematic parameters come into play. The first of these is the “geometric velocity”, also referred to as the “instantaneous transmission ratio.” As the name implies, it characterizes the kinematic correlation between the output and input velocities.

Taking into account the power balance equation for an ideal case, the input power is equal to the output one.

(3) $$W_{output}=W_{input}$$

where $W_{output}=Torque\times angular\;velocity$ and $W_{input}=Force\times velocity$, since the input is related to the muscle length (*l*) and the output to the jaw gape angle (*φ*).

Expressing the powers as stated:

(4) $$\begin{array}{c} T_{output}\cdot \frac{d\varphi }{dt}=F_{input}\cdot \frac{dl}{dt}\\ T_{output}\cdot \frac{\frac{d\varphi }{dt}}{\frac{dl}{dt}}=F_{input} \end{array}$$

The instantaneous transmission ratio is calculated by dividing the differential of the jaw gape angle (*φ*) by the differential of the muscle length (*l*):

(5) $$\varphi^{\prime }=\frac{\;\;\;\frac{d\varphi }{dt}\;\;\;}{\;\;\;\frac{dl}{dt}\;\;\;}=\frac{d\varphi }{dl}$$

A higher value of the geometric velocity indicates a greater velocity amplification, suggesting that the mechanism can convert a slow input movement into a rapid output motion.

Considering the following equation:

(6) $$F_{input}=T_{output}\cdot \varphi^{\prime }$$

A high geometric velocity also results in a reduction in the force or torque exerted by the final element of the mechanism. The geometric velocity is inversely proportional to the force amplification. It is assumed that power dissipations are not present within the mechanism, which would further reduce the output torque.

**Figure 2:**
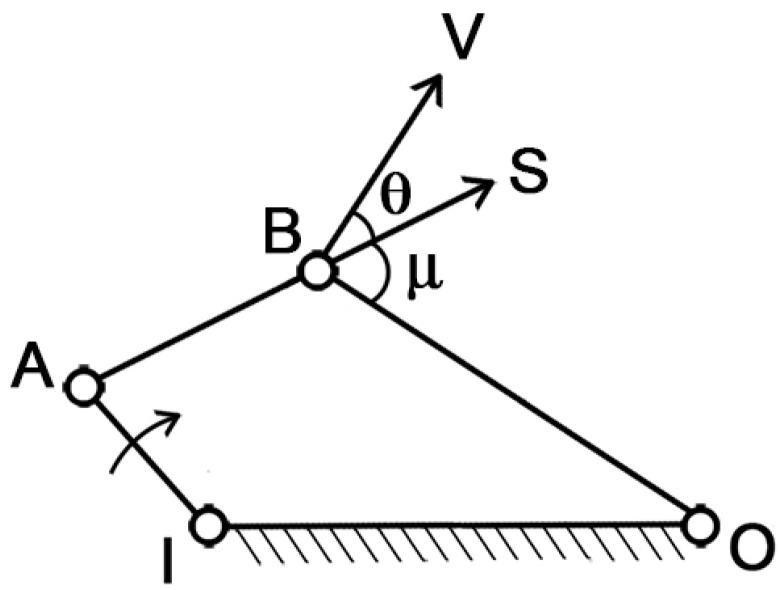
Pressure angle θ in a linkage mechanism. IA is the driver link, BO is the follower link, AB is the coupler, V is the vector of the velocity of point B, and S is the force applied to point B.

To assess the mechanism’s ability to transfer motion from muscle contraction to mouth opening/closing, the “pressure angle” parameter can be introduced. With reference to Figure 2, IA is the driver link, which is the moving element, BO is the follower, AB is the coupler link, and IO is the ground, which is the fixed part. In this simple arrangement, S represents the force applied by the driver indirectly on the follower, while V represents the velocity of the point of application. The pressure angle (θ) is defined as the smaller of the two angles formed by the directions of the force S and the velocity V.

Clearly, as θ increases, the effectiveness of the force S on the follower diminishes. In the extreme case where θ = π/2, the force becomes entirely unsuitable for transmitting the motion. Therefore, the optimal condition is when θ = 0, which maximizes the transmission of motion.

**Figure 3:**
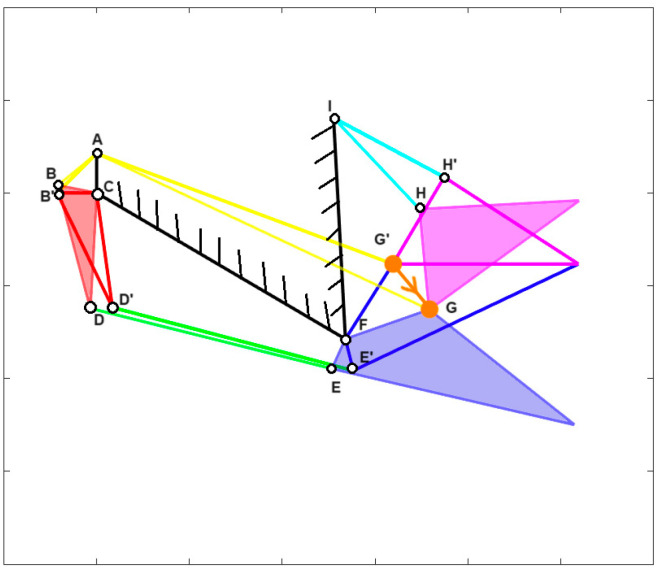
In orange, displacement of the attachment point (G’-G) between upper and lower jaw for *Cheilinus chlorourus*.

Furthermore, for many fishes, the opening and closing of their mouth involve a roto-translation of the jaw. To account for this, a new parameter called “jaw protrusion” (γ) is introduced, which considers the linear displacement of the attachment point of the upper jaw with the lower one. This can be seen in Figure 3, following the orange line G’ to G, for *Cheilinus chlorourus*, where G’ is the closed position, and G the opened one.

With this mechanical model and the defined kinetostatic parameters, the analysis of jaw biomechanics in different species becomes possible. This approach facilitates a more in-depth comparison of how the skeletal mechanisms of these species function, leading to intriguing mechanical insights.

In the next subsection, the jaw anatomy of several different species will be modeled and described using kinematic diagrams.

### 2.2. Kinematic Schemes

In applying the previously described methodology, we consider seven species of bony fish and one species of cartilage fish, each distinct in size and shape. The species are *Cheilinus chlorourus* [21], *Micropterus salmoides* [22], *Eustomias obscurus* [23], the extinct *Dunkleosteus terrelli* [24], *Lepomis macrochirus* [25], *Chlorurus sordidus* [26], and the cartilage fish *Chiloscyllium plagiosum* [27].

The diverse nature of these seven species allows us to analyze different jaw structures, exhibiting variations during both the opening and closing phases, as evident from the mechanisms presented in this section. This selection is crucial to demonstrate how the proposed approach facilitates comparisons among species that differ significantly from one another, making it as general as possible.

**Figure 4:**
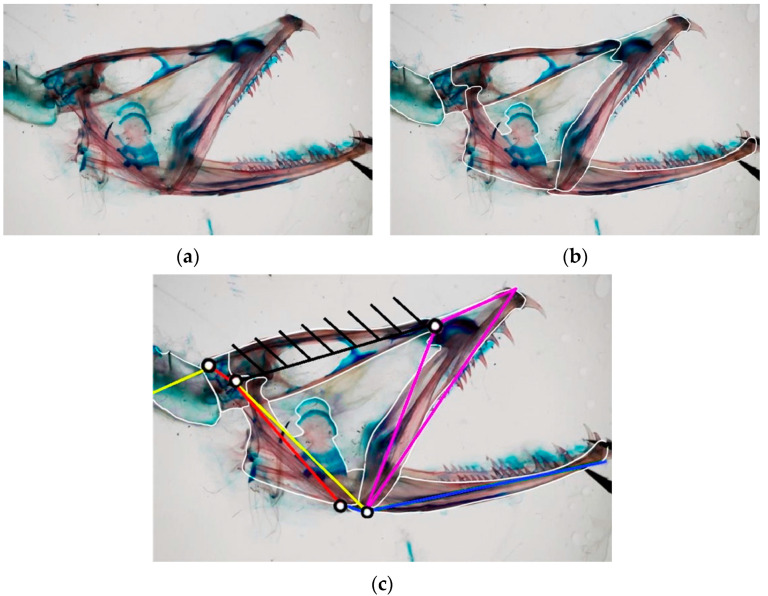
(**a**) Radiography of Eustomias obscurus [29]; (**b**) radiography of Eustomias obscurus with bodies highlighted; (**c**) radiography of Eustomias obscurus with bodies highlighted and corresponding linkage mechanism.

For each species, we conduct an analysis of the jaw anatomy, identifying the muscles responsible for mouth opening and closing movements. This information is then used to create the mechanical model for all the species, depicting them as one or more four-bar linkage mechanisms [28]. As an illustration, Figure 4a showcases the head anatomy of *Eustomias obscurus*, emphasizing its bones. Based on observations of its cranium, the structure can be divided into key links and bodies, as shown in Figure 4b. This step is fundamental in understanding how the structure functions and extracting the linkage mechanism depicted in Figure 4c.

**Figure 5:**
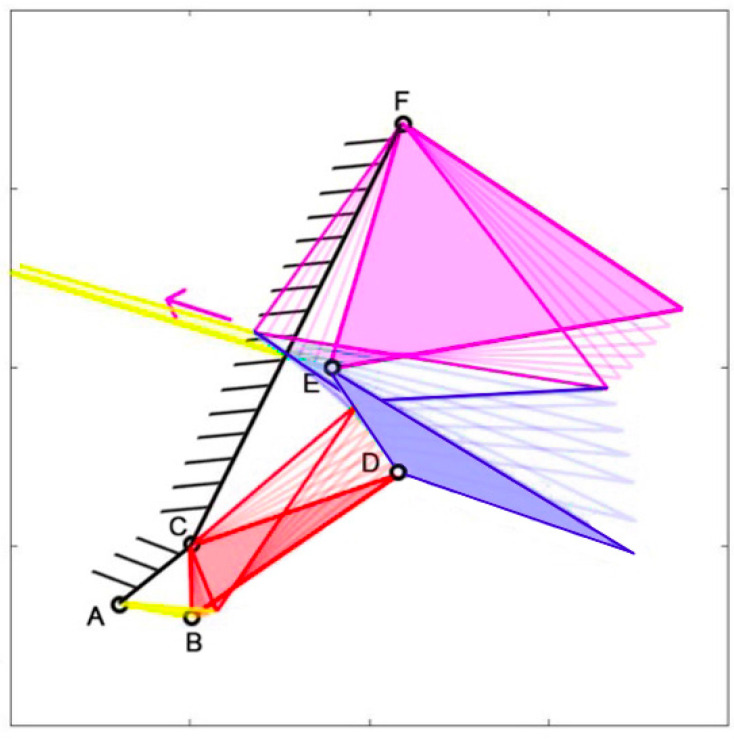
Kinematic scheme of *Chlorurus sordidus* during mouth closing.

The structure can simulate the opening and closing movements of the species’ mouths, as illustrated in Figure 5, which demonstrates the closing mechanism of *Chlorurus sordidus*. The full-colored mechanisms represent the initial state, where the jaws are opened, while the highlighted but empty ones are the final state, with the jaws closed.

Each kinematic scheme and the corresponding graphs are implemented using Matlab. For each species, a table is integrated, which reports the length of the different links. These quantities are scaled with respect to the real case, maintaining the same proportions between them in order to be accurate. This relates to the fact that there is no interest in the size of the species, but on the structure itself. For this reason, the lengths can be considered dimensionless. Then, these quantities have been normalized with respect to the length of the link “r” of the upper jaw. Each structure has the muscles highlighted in yellow, with the arrows showing their contraction, the frame is highlighted in black, and the upper and lower jaws respectively are highlighted in purple and blue.

**Figure 6:**
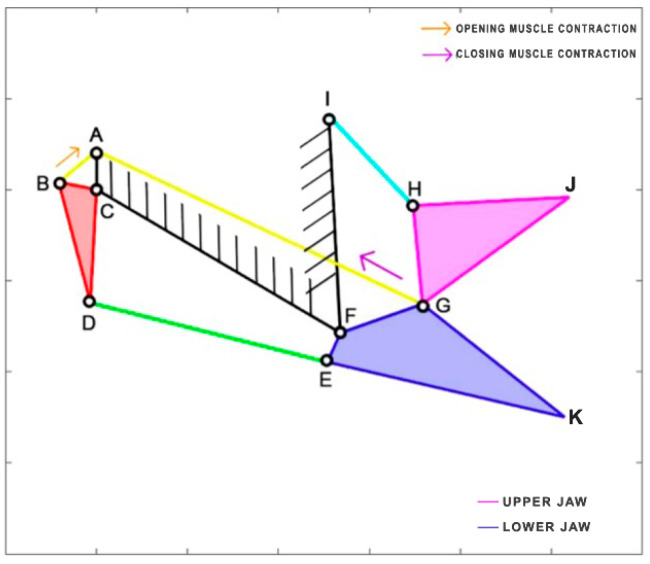
Kinematic scheme of the *Cheilinus chlorourus*.

*Cheilinus chlorourus* belongs to the Labridae family, and it is native to the Indian Ocean and the western Pacific Ocean. This carnivorous fish can reach up to 45 cm in total length. Its feeding mechanism exploits suction feeding, a technique through which it captures preys by generating a flow of water into a rapidly expanding mouth cavity [30]. It feeds mainly on benthic invertebrates such as mollusks and crustaceans. The cranial osteology and muscles of *Cheilinus chlorourus* were studied by Westneat [31] to understand its feeding mechanism. The opening and closing of the mouth are controlled by specific muscles: the levator operculi for opening and three adductor muscles for closing [31]. The three adductor muscles are simplified into a single element. From the observation of its cranial anatomy, two mechanisms are derived: the anterior jaws linkage (FGHI) and the opercular linkage (CDEF), as depicted in Figure 6. During the mouth’s opening, the contraction of the levator operculi muscle induces a clockwise rotation of the red link, which serves as the input for the opercular linkage. The opercular linkage, located at the bottom left, consists of an input link (CD), a fixed link above (CF), the coupler link (DE) at the bottom, and the output link (EF) on the right. The output of the opercular linkage then serves as the input for the anterior jaws linkage, resulting in the opening of the fish’s mouth. In the closing phase, the input is directly connected to the jaws, representing the overall adductor muscle. Contraction of this muscle causes the jaws to close. Table 1 reports the length of each link.

**Figure 7:**
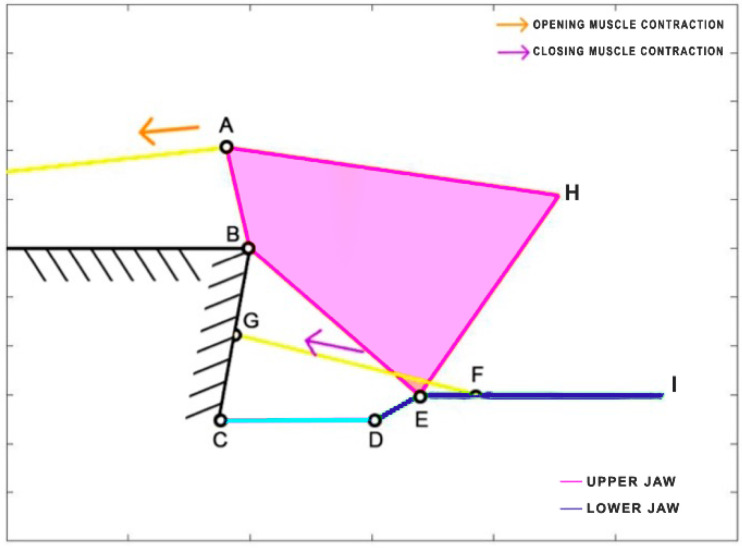
Kinematic scheme of *Micropterus salmoides*.

*Micropterus salmoides*, a carnivorous freshwater fish, belongs to the Centrarchidae family and is native to the United States, Canada, and Mexico. Due to its popularity as a game fish, it has been widely introduced and is now found worldwide. Adult individuals primarily feed on fish, crayfish, and frogs, while the younger ones consume crustaceans, insects, and small fish. Interestingly, cannibalism can sometimes occur within this species. With a maximum length of 97 cm, it is a relatively large fish. During the opening and closing phases of its mouth, it relies on two fundamental muscles: the epaxial muscle (starting from point A) and the sternohyoid muscle (attached to the lower jaw FG). Notably, the epaxial muscle is remarkably long, extending throughout the entire body of the fish [32]. To better understand its mechanics, the kinematic scheme is developed based on the analysis published by Olsen et al. [33], representing it as a four-bar linkage mechanism (Figure 7). Table 2 reports the length of each link.

**Figure 8:**
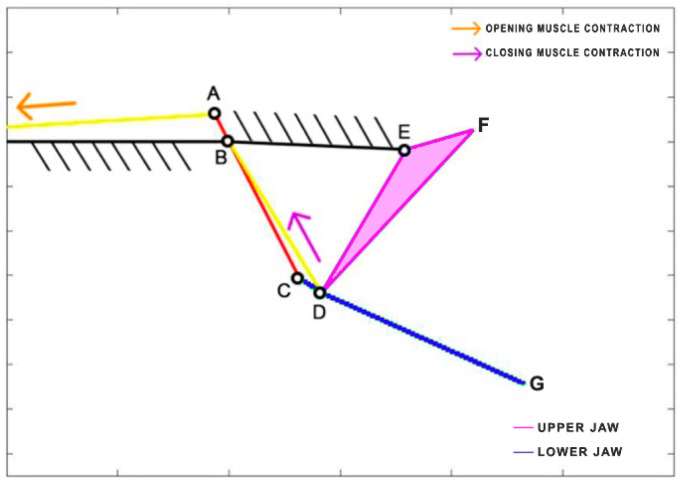
Kinematic scheme of *Eustomias obscurus*.

*Eustomias obscurus* is a deep-sea carnivorous fish belonging to the Stomiidae family, primarily found in the oceanic depths of the Atlantic Ocean. Typically measuring around 15 cm in length, it can reach up to 26 cm. These fish are apex predators, characterized by their formidable jaws filled with fang-like teeth. Remarkably, they possess the unique ability to hinge their neurocranium and upper-jaw system, enabling a jaw opening of more than 100 degrees. This remarkable flexibility allows them to consume prey that is often 50% larger than their own standard length. The mouth opening is driven by the epaxial muscle (from point A, extending towards the left), once again covering the entire body of the species. This muscle’s contraction, combined with an additional head joint, allows the jaw to open up to an impressive 120 degrees [34]. The kinematic scheme (Figure 8) for this species was developed by Burgess [11], illustrating how the contraction of the epaxial muscle causes the rotation of the input link in the four-bar linkage mechanism, ultimately resulting in the rotation of the output link, which produces the mouth opening motion. Table 3 reports the length of each link.

**Figure 9:**
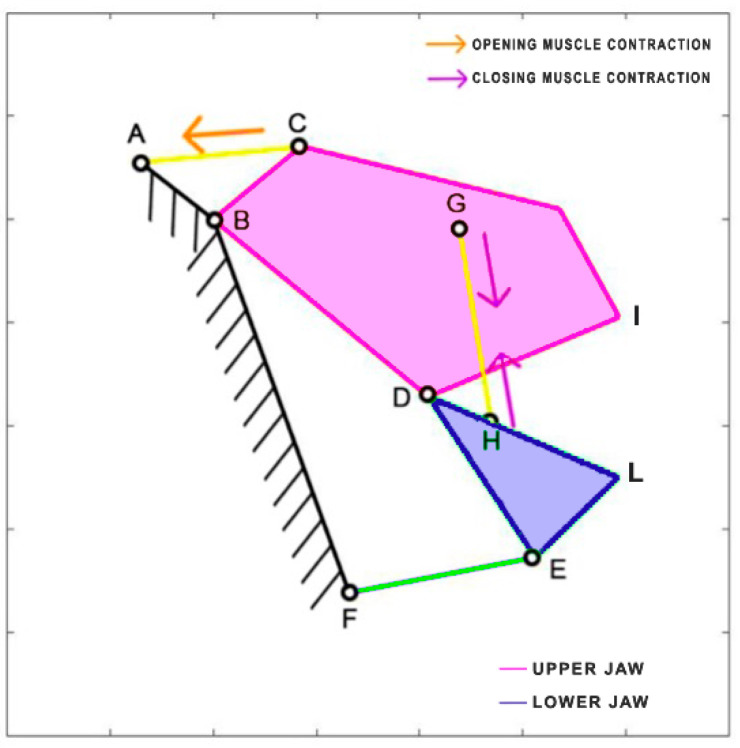
Kinematic scheme of *Dunkleosteus terrelli*.

*Dunkleosteus terrelli*, an extinct placoderm fish, holds the distinction of being the largest armored jawed fish, reaching an impressive length of up to 8.79 m. This ancient carnivorous fish possessed the unique ability to rapidly open and close its jaw for suction feeding, while also boasting an incredibly high biting force. Anderson and Westneat [35] developed a kinematic model, utilizing a single four-bar linkage mechanism. In this model, the opening muscle is represented by the epaxialis (AC), while the closing muscles are the adductor mandibulae (GH), which are simplified as a single element in the scheme presented in Figure 9. Table 4 reports the length of each link.

**Figure 10:**
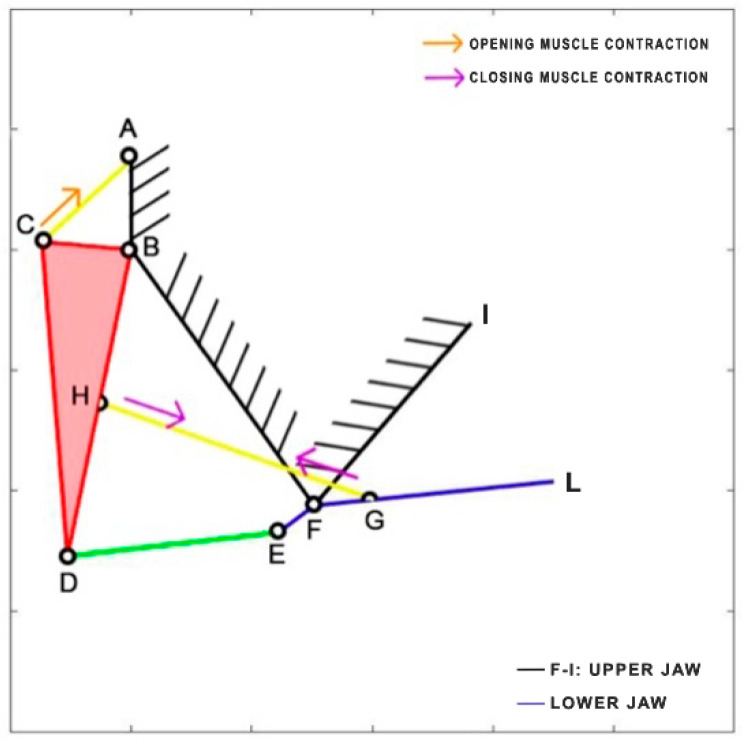
Kinematic scheme of *Lepomis macrochirus*.

*Lepomis macrochirus* is a carnivorous freshwater fish belonging to the family Centrarchidae, native to North America. Its length can reach 30 cm. Similar to *Cheilinus chlorourus*, the levator opercula (CA) muscles are responsible for mouth opening, while two adductor muscles come into play during the closing phase [36]. The kinematic scheme [37] is depicted in Figure 10, where, once again, the closing muscles are simplified into a single element (HG) directly connected to the lower jaw. Unlike the other species, *Lepomis macrochirus* has a fixed upper jaw. Table 5 reports the length of each link.

**Figure 11:**
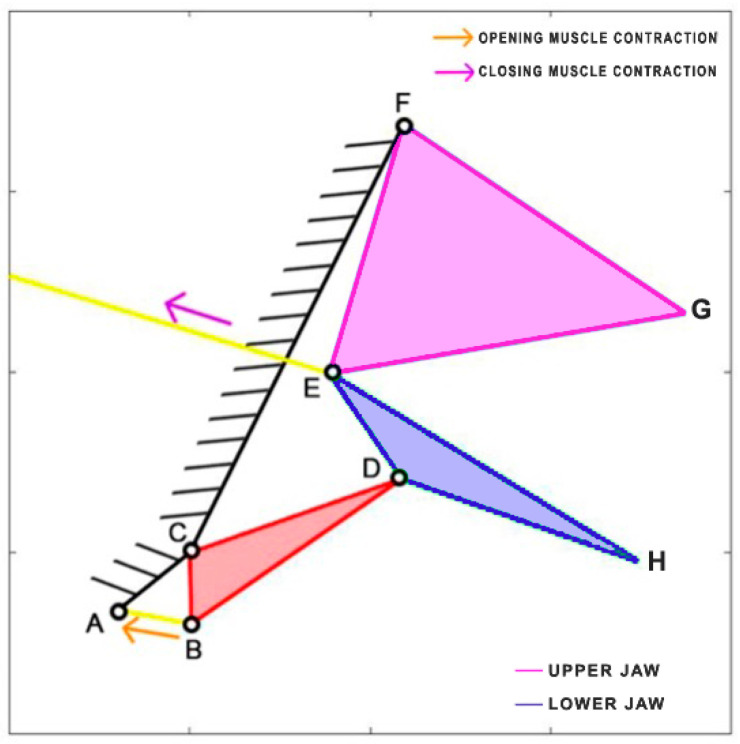
Kinematic scheme of *Chlorurus sordidus*.

*Chlorurus sordidus*, a member of the Scaridae family, is the only herbivorous species considered in this study. It is known for its coral-biting behavior, consuming the symbiotic microalgae from coral polyps. This species is widespread in the tropical waters of the Indo-Pacific region and can reach a maximum length of 40 cm. Belonging to the same order as *Cheilinus chlorourus*, the Labriformes, *Chlorurus sordidus* employs the levator posterior muscle and the adductor mandibulae for the opening and closing of its jaws, even though their sizes differ significantly from the labrids [38]. In the kinematic scheme presented in Figure 11, the contraction of the levator posterior (AB) serves as the input of the four-bar linkage mechanism during the opening phase. On the other hand, the single overall adductor muscle (starting from point E) is connected to the jaws, and its contraction results in the closing of the mouth. Table 6 reports the length of each link.

**Figure 12:**
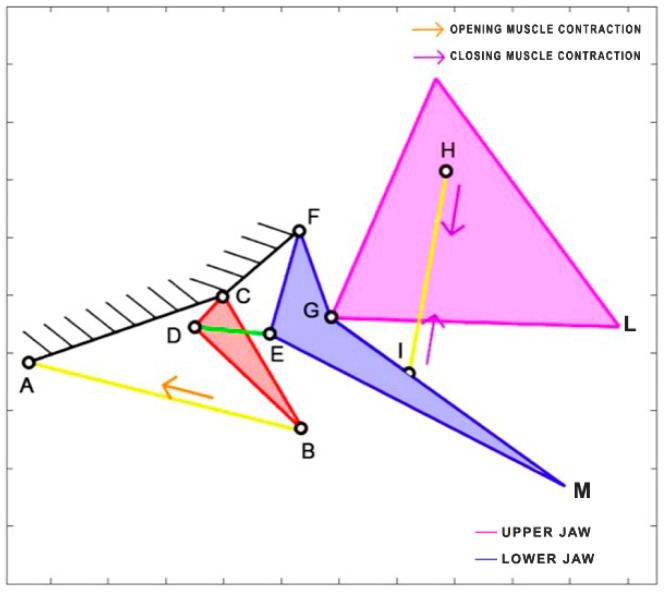
Kinematic scheme of *Chiloscyllium plagiosum*.

*Chiloscyllium plagiosum* is a cartilage fish belonging to the Hemiscylliidae family, inhabiting the Pacific Ocean. This carnivorous shark prefers to feed at night, preying on small fish and invertebrates. It can reach a length of up to 93 cm. The kinematic scheme derived by Ramsay and Wilga [39] is illustrated in Figure 12. In this scheme, the mouth opening is facilitated by the contraction of the coracohyoideus and coracoarcualis muscles, which are depicted as a single element (AB). Similar to *Dunkleosteus terrelli*, the adductor muscles (HI) come into action during the closing phase. Table 7 reports the length of each link.

## 3. Kinetostatic Analysis

With the chosen fish species and mechanical parameters in place, the next step involves analyzing the kinematic diagrams introduced in Section 2.2. The analysis will focus on several aspects, including the range of the jaw’s gape angle, the velocity amplification, the jaw protrusion during motion, and the efficiency of the linkage mechanism in transmitting the motion.

As explained, the first analysis performed is relative to the gape angle of the jaw. Not only is the maximum size of each species considered but also its behavior during the whole motion compared to the input. Two distinct analyses are carried out corresponding to the opening and closing phase of the jaw, as the input muscle contraction (*ν* and *ρ*).

The first parameter aims to compare how the biological structure converts the linear motion of the muscle into jaw rotation, disregarding the length of the muscle. On the other hand, the second parameter accounts for the size of the muscle and concentrates on the input actuation.

**Figure 13:**
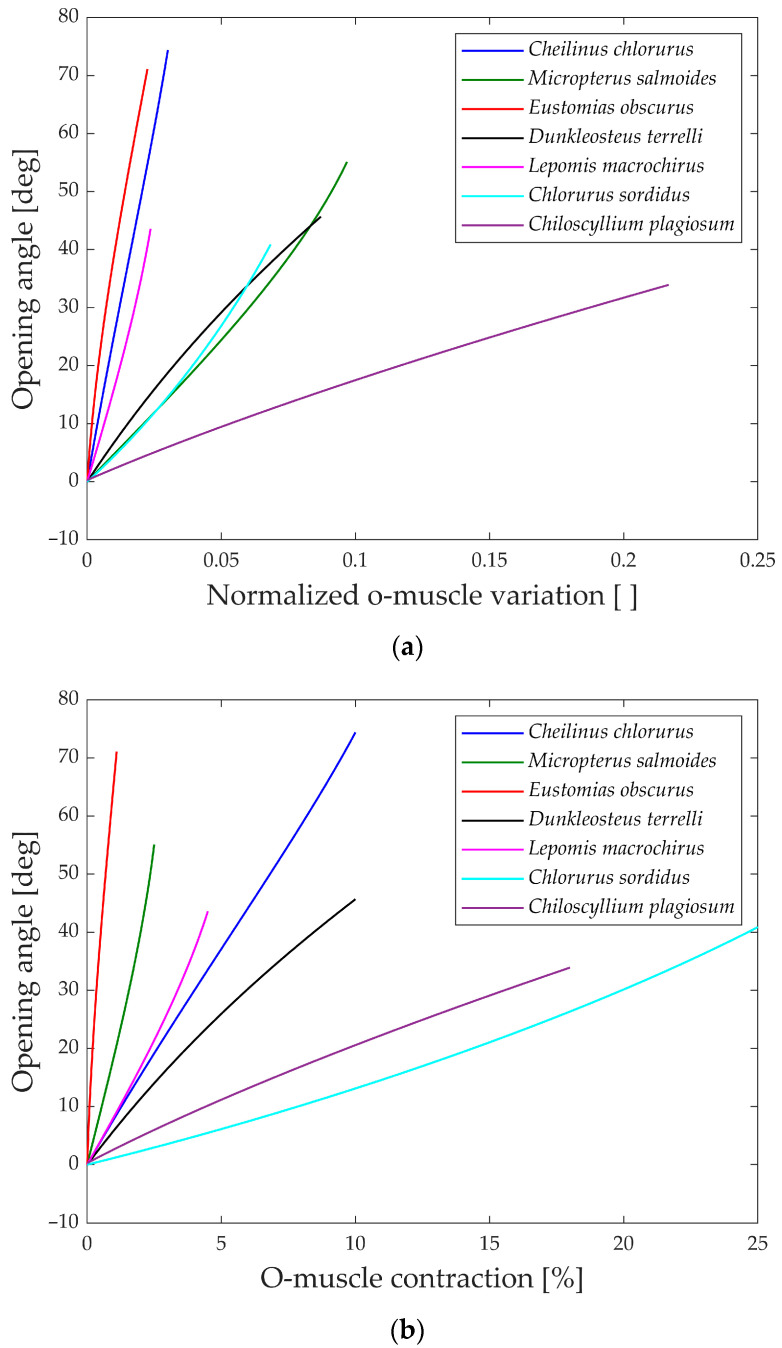
(**a**) Opening angle with normalized muscle variation; (**b**) opening angle with percentual muscle contraction.

In Figure 13a, the gape angle during the opening phase is depicted in relation to the normalized contraction of the opening muscle.

All the species initiate the jaw movement with a fully closed position and proceed to open it by contracting the opening muscle. Notably, species like *Eustomias obscurus*, *Cheilinus chlorourus*, and *Lepomis macrochirus* exhibit steeply sloped trends, indicating that their structures possess amplification mechanisms, enabling them to convert a small linear displacement into a substantial jaw rotation. On the other hand, *Micropterus salmoides*, *Dunkleosteus terrelli*, *Chlorurus sordidus*, and *Chiloscyllium plagiosum* display curves with lower gradients, suggesting that their linkage mechanisms do not significantly amplify the motion, unlike the first set of species. However, the gape angle plots in Figure 13b reveal changes in position for some species: *Micropterus salmoides* now has a steeper slope, while *Cheilinus chlorourus* and *Lepomis macrochirus* exhibit slightly more horizontal trends. Additionally, *Chlorurus sordidus* becomes much more horizontal. These shifts are attributed to the size of their opening muscles; larger muscles result in smaller percentual contractions for the same linear contraction. This suggests that some species achieve motion amplification through their linkage mechanism, while others rely on longer muscles for the same effect. In contrast, the shark *Chiloscyllium plagiosum* shows horizontal lines in both graphs, indicating a lack of both a bone structure that amplifies motion and a long opening muscle.

**Figure 14:**
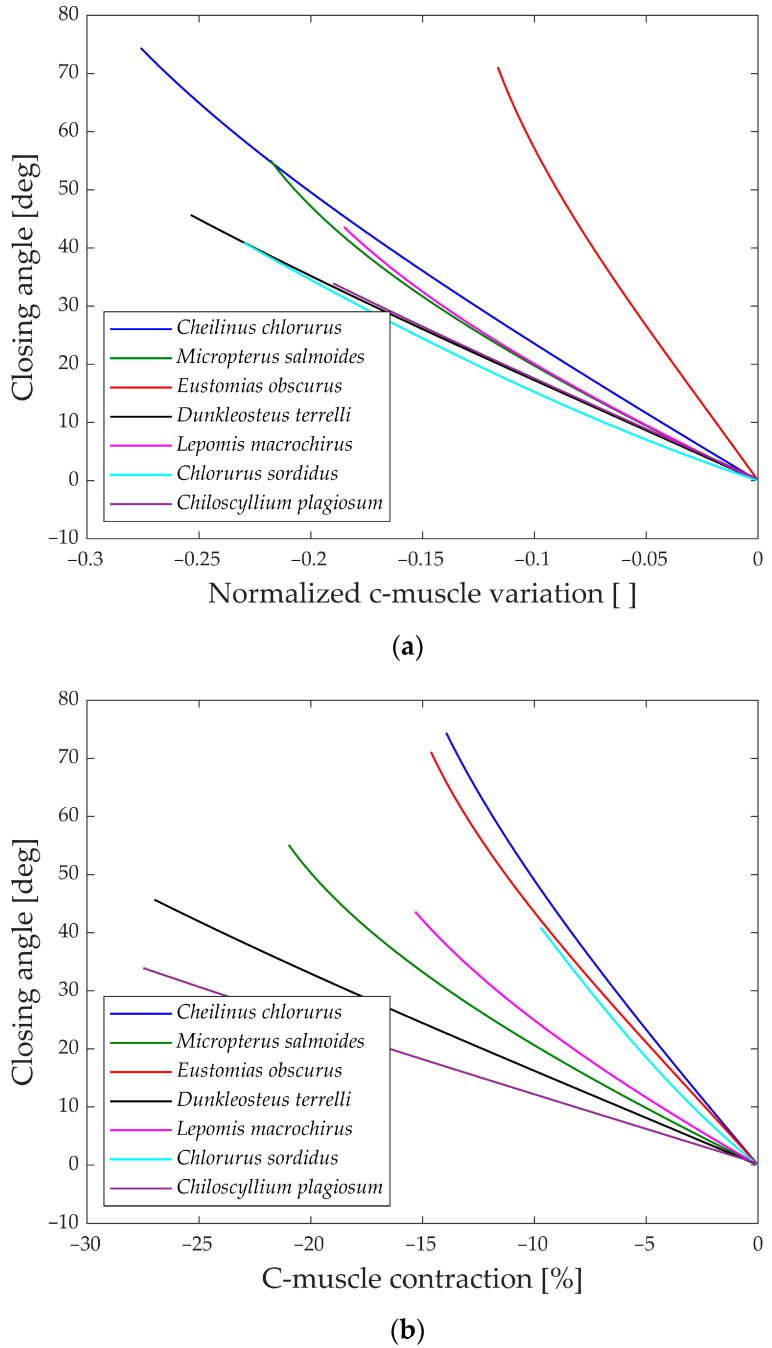
(**a**) Closing angle with normalized muscle variation; (**b**) closing angle with percentual muscle contraction.

Moving to Figure 14a,b, the gape angle variation during the closing phase is shown in relation to the contraction of the closing muscle. The closing mechanism differs significantly from the opening one as the models start with an open jaw (left part of the graphs) and then proceed to close it until the gape angle reaches zero (right part of the graphs).

An intriguing observation in both the opening and closing graphs is that, despite the presence of complex linkage mechanisms, the gape angle exhibits a relatively linear behavior in all the species considered.

To gain further insights, the analysis of the geometric velocity of the linkage mechanisms provides valuable observations. This mechanical parameter helps estimate how a linkage mechanism converts input velocity into output velocity.

**Figure 15:**
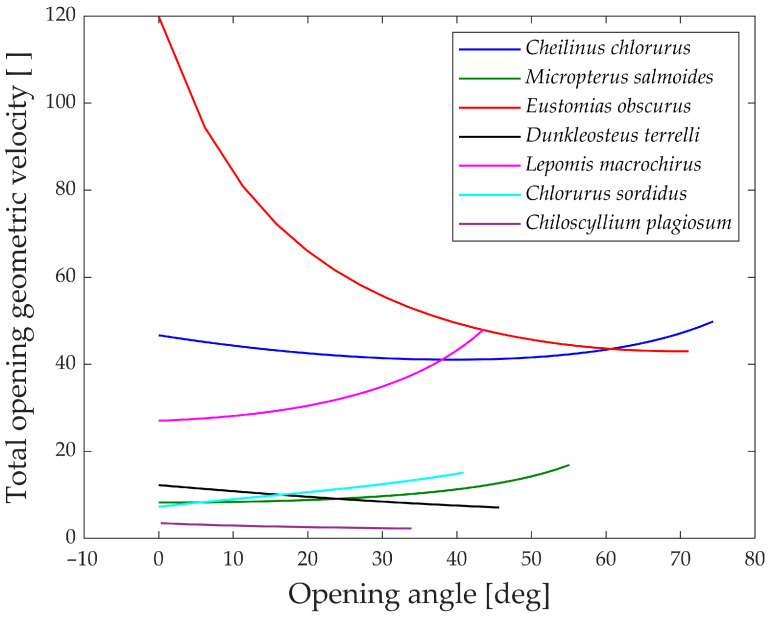
“Geometric velocities” during opening.

In Figure 15, the “geometric velocities” during the opening phase of the jaw are depicted. Notably, every species exhibits a horizontal pattern, reaffirming the linear relationship between input and output.

The only exception is *Eustomias obscurus*, which shows a peak at the beginning of the motion when the upper and lower jaws are fully closed. This exceptional value is attributed to the alignment of two links in the mechanism, resulting in significant velocity amplification from input to output. However, it also indicates that opening the jaw within this range requires considerable force, making the initial degrees of the opening phase quite challenging. A possible explanation for this unique configuration is that in reality, Eustomias obscurus keeps its jaw partially open most of the time, thus, never initiating the opening of the jaws from 0°. This species possesses very long and sharp teeth, allowing it to capture prey without fully closing its jaw.

Before delving into the values of the different geometric velocities among the seven species, it is essential to remember that all species, except *Chlorurus sordidus*, employ suction feeding, necessitating a rapid mouth opening.

As observed, some species achieve opening velocity amplification through their linkage mechanisms: *Eustomias obscurus*, *Cheilinus chlorourus*, and *Lepomis macrochirus* stand out in this regard. As previously explained, *Micropterus salmoides* lacks amplification through its linkage mechanism, resulting in limited geometric velocity, significantly lower than the first group of species. However, it compensates for this by utilizing the suction feeding mechanism effectively. Its long muscle allows for high-speed contractions, leading to substantial opening velocity.

*Chiloscyllium plagiosum* exhibits an intriguing behavior; it also possesses a low geometric velocity but, unlike *Micropterus salmoides*, lacks a long opening muscle. As a result, it cannot achieve high velocity through either amplification or a faster input. Instead, it utilizes a unique mechanism that involves locking the jaw via a specific alignment of links and muscles. The muscle contracts without moving the jaw until it is fully loaded, at which point the jaw is unlocked, releasing the stored energy and achieving very high speeds.

As anticipated, *Chlorurus sordidus* also demonstrates a very low geometric velocity, as it is an herbivorous fish that does not require suction feeding.

While the comparison of only seven species has its limitations, it has already revealed three completely distinct solutions to achieve rapid jaw opening.

**Figure 16:**
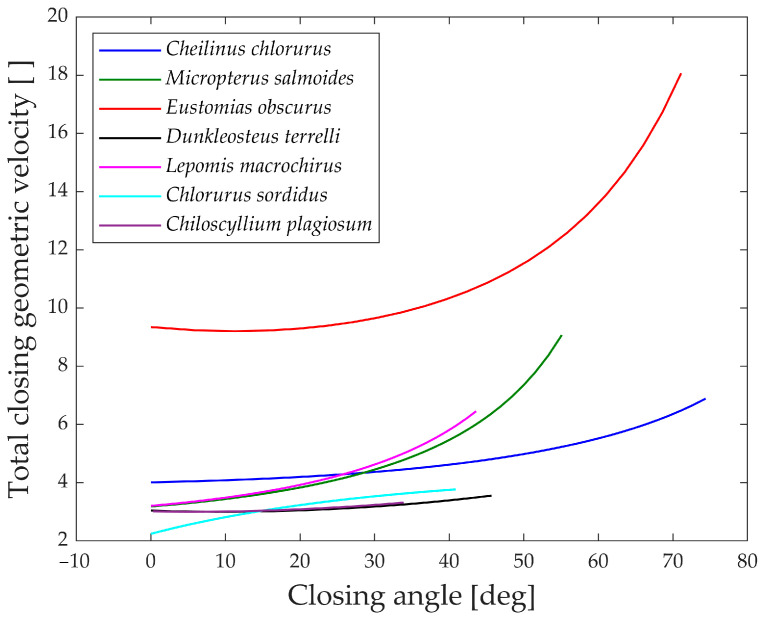
“Geometric velocities” during closing.

In Figure 16, the “geometric velocities” during the closing phase of the jaw are illustrated. Generally, all studied species exhibit a slight decrease in geometric velocity as the jaw closes, indicating that speed amplification is at its minimum at this point. This suggests that high-velocity amplification is most advantageous when the jaw is fully open, enabling swift prey capture in the initial moments. Another potential explanation is that, due to kinetostatic duality, achieving the highest force is more efficient when the jaw is closed.

Upon examining the values, *Dunkleosteus terrelli*, *Chiloscyllium plagiosum*, and *Chlorurus sordidus* are observed to have the lowest geometric velocities. Notably, these species do not close their jaws solely to prevent food leakage but also use their bite to cut and crush prey. While the first two species bite their prey, the last one is an herbivore but requires a powerful bite to break down coral. For these three species, having a low geometric velocity during the closing phase corresponds to higher transmitted forces to the jaw, contributing to their ability to achieve high biting forces.

**Figure 17:**
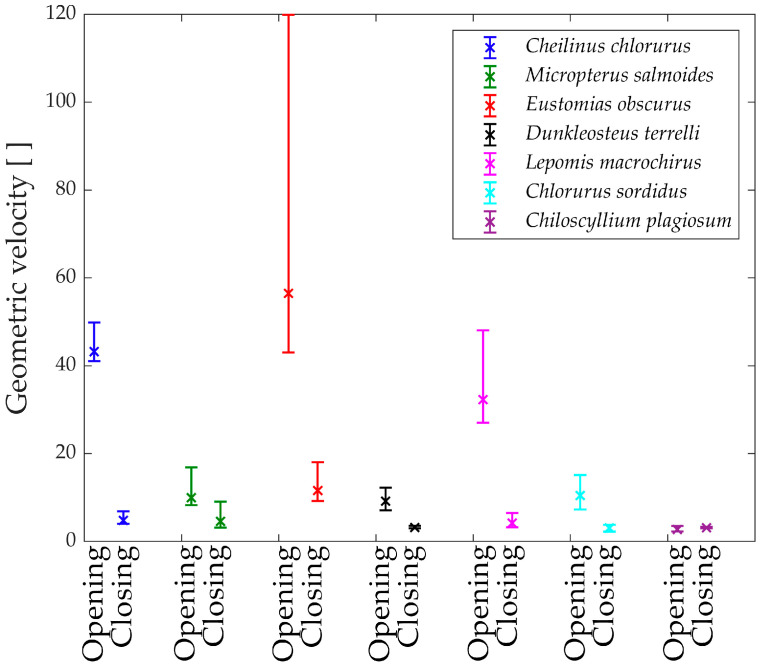
Variation of the “Geometric velocities”. Opening on the left, closing on the right.

Figure 17 depicts the “geometric velocities” from the opening phase to the closing phase in all seven species. In each case, the closing geometric velocity is lower than the opening one. This difference can be attributed to the species’ need to attain higher speeds during the opening phase to facilitate suction feeding. For these species, it is crucial to be swift in opening the jaw for efficient prey capture.

Species that do not exhibit a significant difference between opening and closing velocities are those that achieve high velocities through other means or, in the case of *Chlorurus sordidus*, do not rely on suction feeding.

**Figure 18:**
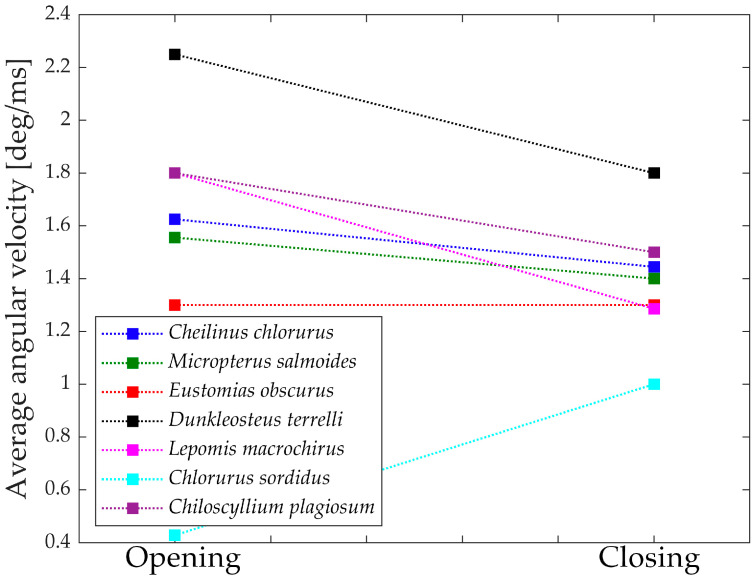
Average angular velocities from literature. Opening on the left, closing on the right.

Figure 18 presents the measured mean velocities from the literature [31,32,33,34,35,36,37,38,39,40], which align perfectly with the numerically obtained results. Interestingly, the proposed mechanical approach goes beyond what biologists typically measure, as it provides additional information allowing for the study of forces and speeds throughout the entire motion.

**Figure 19:**
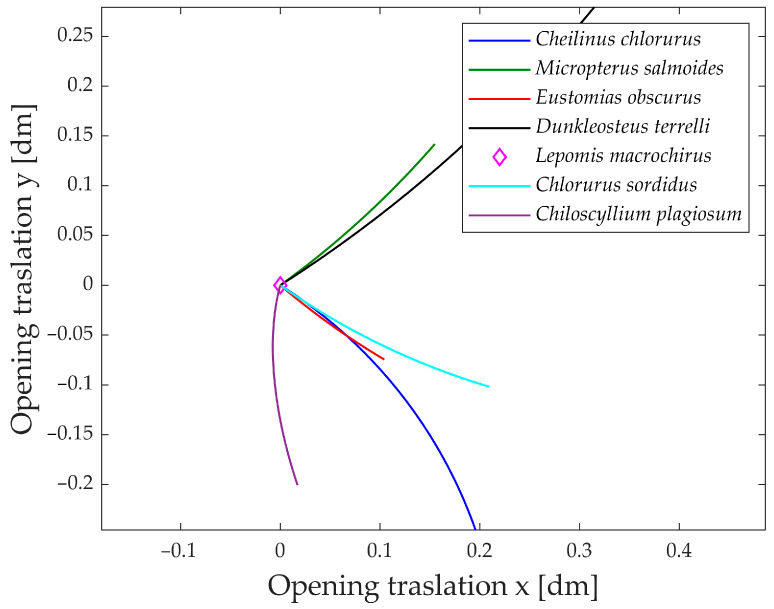
Shift during opening.

An intriguing aspect to analyze is the jaw’s movement in the plane during its opening. Figure 19 illustrates the jaw protrusion throughout the entire motion. Nearly all species exhibit a translation along the x-axis, indicating that they extend their mouth forward during feeding. This feature proves highly advantageous during prey capture, as the extended jaw moves closer to the prey, enhancing the effectiveness of suction feeding [41].

Based on the translation along the y-axis, the species can be grouped into two distinct categories. The first group comprises *Dunkleosteus terrelli* and *Micropterus salmoides*, both exhibiting an upward elevation of the jaw during opening. In contrast, the second group, consisting of *Cheilinus chlorourus*, *Eustomias obscurus*, *Chlorurus sordidus*, and *Chiloscyllium plagiosum*, displays a downward motion of the jaw.

An interesting observation is that species in the first group have their opening muscle directly connected to the upper jaw, while the lower jaw moves as a consequence of the upper jaw’s rotation. In contrast, species in the second group have their opening muscle connected through a kinematic chain to the lower jaw first. Consequently, the upward or downward translation of the jaw is determined by which part is directly rotated and which one is subsequently moved.

**Figure 20:**
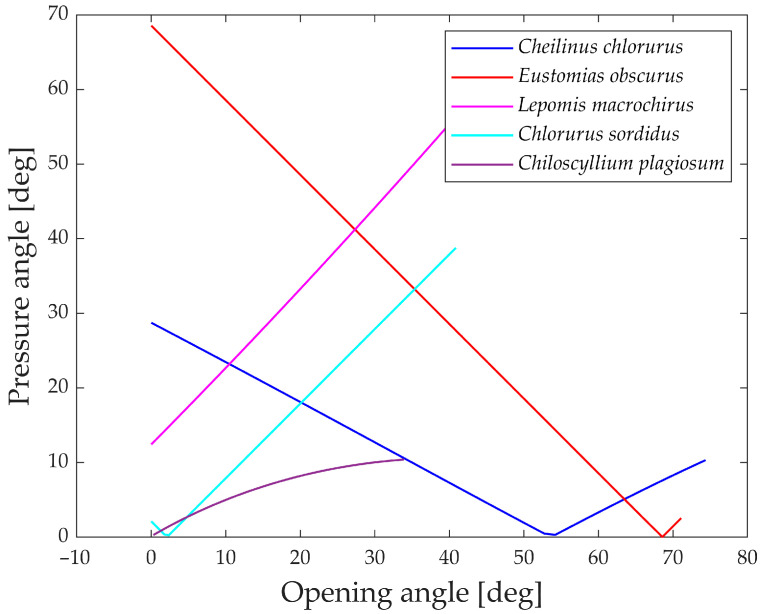
Pressure angle for the linkage mechanism.

The pressure angle serves as an indicator of the linkage mechanism’s effectiveness in transmitting input force to output motion. Figure 20 illustrates the pressure angle for five species, as it proves to be a relevant parameter for their structures compared to the other two.

An ideal transmission of motion occurs when the pressure angle is close to 0°, while a value higher than 45° to 50° typically indicates that the mechanism requires a considerable force to complete the motion.

All five species showcased in the graph exhibit values below this threshold, indicating efficient transmission of force. The only exceptions are *Eustomias obscurus* during the first 20° of jaw opening and *Lepomis macrochirus* when its jaw is fully open. In both cases, the pressure angle is high due to an alignment of links that results in an inefficient force transmission. However, as mentioned previously, *Eustomias obscurus* avoids this unfavorable condition by never completely closing its mouth, while *Lepomis macrochirus* may not typically open its jaw to such an extent.

**Figure 21:**
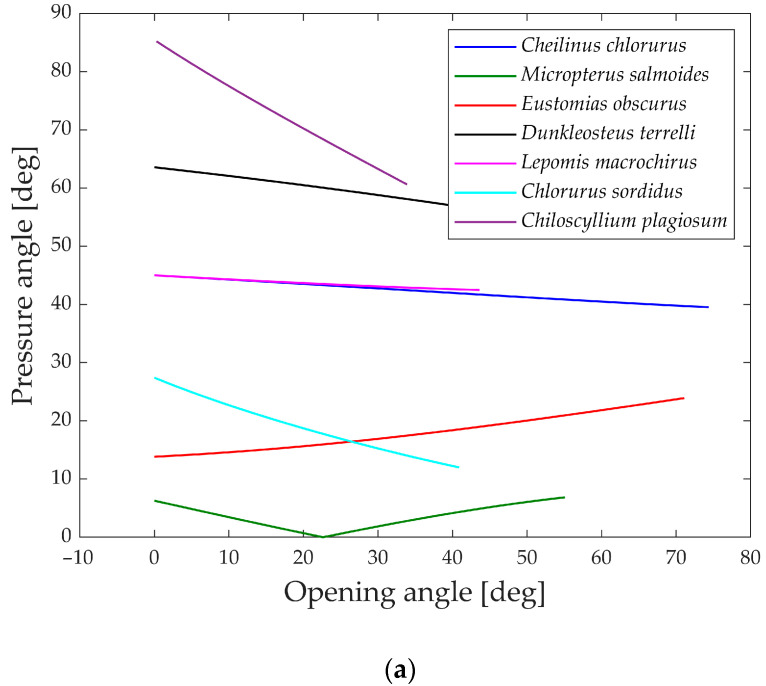

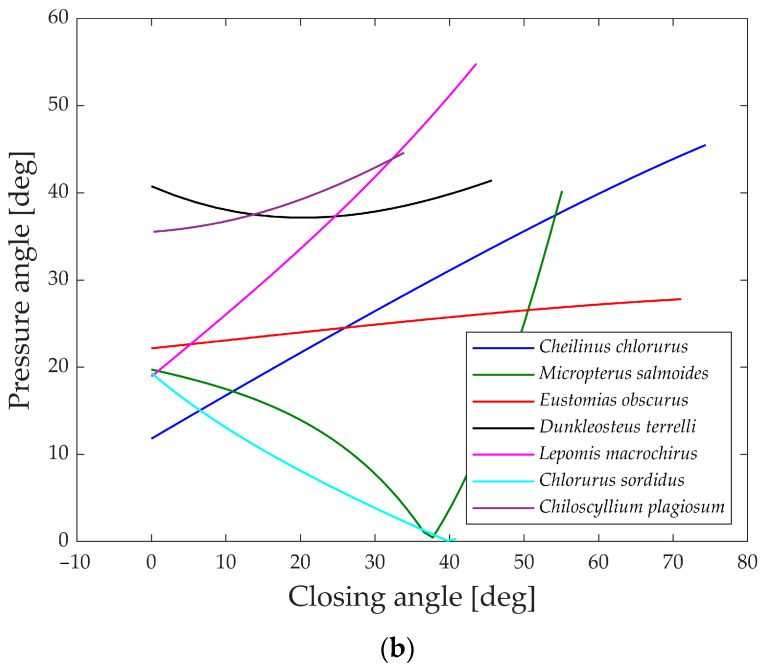
Pressure angle in muscle’s attachment area. (**a**) During opening; (**b**) during closing.

In Figure 21a,b, the pressure angles in the muscle’s attachment area are depicted during the opening and closing phases. A smaller angle indicates a better alignment of the muscle with the link motion, resulting in more efficient transmission.

During the opening phase, five species maintain pressure angles below 50°, achieving a reasonably effective transmission of force. However, *Dunkleosteus terrelli* and *Chiloscyllium plagiosum* deviate from this pattern, exhibiting higher values. Particularly, *Chiloscyllium plagiosum* begins with a pressure angle close to 85°, indicating that when the jaw is completely closed, the action force of the opening muscle is perpendicular to the direction of motion of the first link. While this might typically be deemed unfavorable for transmission, it showcases how *Chiloscyllium plagiosum* employs a snapping mechanism to achieve high opening velocity. With a pressure angle close to 90°, it can contract the muscle and store energy while the jaw remains stationary, releasing it in an instant for rapid jaw movement.

In contrast, during the closing phase, all the analyzed species exhibit pressure angles below 50°, allowing for a decent transmission of muscle force to the closing mechanism. Once again, the values of *Lepomis macrochirus* at the end of the motion can be attributed to its smaller opening range.

## 4. Conclusions

The biological realm offers a wealth of fascinating behaviors and mechanisms, particularly intriguing from an engineering perspective, as they may inspire innovative solutions in the field of robotics.

The proposed quantitative approach is founded on mechanical parameters, allowing engineers to compare vastly different species, thereby providing them with a method to select and analyze the most suitable options for developing bio-inspired solutions.

By modeling the jaws of seven fish species as linkage mechanisms and examining mechanical elements like geometric velocities and pressure angles, this comparison not only validates unique biological traits, such as feeding techniques and dietary preferences, but also acts as a guide for pinpointing the most suited species according to specific requirements.

Looking ahead, this approach holds potential for broader applications across various species, enabling the creation of bio-inspired solutions to meet engineering requirements. New indices, based on the biomechanics approach, are able to describe some aspects of the behavior of the fish jaws that could be useful for the design, for example, of a bio-inspired manipulator. The analysis conducted offers interesting insight useful during the development of the gripper, and the biological behaviors of the species, such as their feeding methods, can be exploited through the mechanical parameters introduced. For example, the jaw protrusion of the species’ mouth can be harnessed for gripping objects while the manipulator is moving, or, conversely, it must be considered when a precise grip is essential. Regarding the gape angle, meaning how much the jaws can open, it would be the maximum size of objects the gripper can grasp. A good value for the pressure angle, which indicates how well the motion is transmitted in the linkage mechanism, means that the structure is optimized, so it will require less force in order to transmit the motion, since the input is mostly transformed into the output.

A natural progression involves transitioning from the purely planar analysis presented in this study to spatial analyses, leading to the realization of manipulators capable of moving objects in three-dimensional space. With more complex designs entailing an increasing number of degrees of freedom, sophisticated control strategies can be developed to enhance functionality and versatility.

In conclusion, this quantitative engineering approach opens exciting avenues for integrating biological insights into robotic designs, fostering advancements and innovation in the field of bio-inspired robotics.

## Figures and Tables

**Table 1:** Length of links for *Cheilinus chlorourus*.

*Cheilinus chlorourus*	
G–J (r)	470
A–C	100
B–C	100
B–D	322.02
C–D	292.75
D–E	630.63
E–F	82.462
C–F	730.62
F–G	224.72
G–H	255.54
H–I	317.65
F–I	560.8
E–K	630.71
G–K	470
H–J	404.97
A–B (muscle)	141.42

**Table 2:** Length of links for *Micropterus salmoides*.

*Micropterus salmoides*	
F–H (r)	778.97
B–	3000
A–B	330
A–H	1073.4
B–E	716.1
D–E	158.11
C–D	503.29
B–C	559.02
E–I	778.97
o_muscle	3018.1

**Table 3:** Length of links for *Eustomias obscurus*.

*Eustomias obscurus*	
D–F (r)	1442
B–	3000
A–B	199.25
B–C	996.24
C–D	170
D–E	1078.1
B–E	1151.1
D–G	1442
E–F	452.77
o_muscle	2946.1

**Table 4:** Length of links for *Dunkleosteus terrelli*.

*Dunkleosteus terrelli*	
D–I (r)	607.98
A–B	273.64
B–C	335.74
C–M	782.41
I–M	363.61
B–D	820.27
D–E	569.67
E–F	558.8
B–F	1170.5
E–L	339
D–L	607.98
A–C (muscle)	529.83
C–I	1062.3

**Table 5:** Length of links for *Lepomis macrochirus*.

*Lepomis macrochirus*	
F–I (r)	659.47
A–B	245
B–C	245
C–D	862.28
B–D	855.86
D–E	580.78
E–F	121.66
B–F	860.23
F–L	659.47
A–C (muscle)	346.48

**Table 6:** Length of links for *Chlorurus sordidus*.

*Chlorurus sordidus*	
E–G (r)	506.36
A–C	128.06
B–C	99.539
B–D	359.18
C–D	315.63
E–D	179.18
E–F	363.74
C–F	670.82
D–H	350.46
E–H	506.36
F–G	475.64
A–B (muscle)	138.52

**Table 7:** Length of links for *Chiloscyllium plagiosum*.

*Chiloscyllium plagiosum*	
G–L (r)	1328.5
A–C	951.89
B–C	730.82
B–D	700.07
C–D	200
D–E	350
E–F	500
C–F	460.98
E–M	1529.7
G–M	1328.5
F–G	430.12
G–N	1204.2
N–L	1423
A–B (muscle)	1600
G–H	832.17

## Data Availability

The data presented in this study are contained within the article.

## Nomenclature

**Symbol**	**Description**
*ν*	Normalized contraction
*ρ*	Percentual contraction
*φ*′	Geometric velocity
θ	Pressure angle
γ	Jaw protrusion
